# The effect of exogenous 24‐epibrassinolide pretreatment on the quality, antioxidant capacity, and postharvest life of wucai (*Brassica campestris* L.)

**DOI:** 10.1002/fsn3.2075

**Published:** 2021-01-23

**Authors:** Lingyun Yuan, Libing Nie, Qiang Ji, Yushan Zheng, Liting Zhang, Shidong Zhu, Jinfeng Hou, Guohu Chen, Chenggang Wang

**Affiliations:** ^1^ College of Horticulture Vegetable Genetics and Breeding Laboratory Anhui Agricultural University Hefei China; ^2^ Provincial Engineering Laboratory for Horticultural Crop Breeding of Anhui Hefei China; ^3^ Wanjiang Vegetable Industrial Technology Institute Maanshan China

**Keywords:** 24‐epibrassinolide, antioxidant capacity, *Brassica campestris* L., postharvest, quality, storage

## Abstract

The quality of green leafy vegetables is easily lost during the postharvest period. The effect of exogenous 24‐epibrassinolide (EBR) pretreatment on the quality of wucai was evaluated in the present study. Wucai plants were sprayed twice with 0.1 μM EBR before harvesting. Two storage temperatures were tested: 25°C and 4°C. At 4°C, EBR pretreatment significantly delayed the degradation of the pigment and plant water loss. Furthermore, we measured the activity of key enzymes of the ascorbic acid (AsA)‐glutathione (GSH) cycle, the content of the main metabolites, and the expression of the AsA metabolism‐related genes in leaves. The results indicated that all three plants showed stronger antioxidant capacity after EBR pretreatment. At 4°C and 25°C, the storage time of wucai was 20 days and 7 days after EBR treatment, while the samples could be stored for 14 days and 4 days without EBR treatment application, respectively. At 4°C, the nutritional properties of wucai pretreated with EBR, such as total free amino acids, total soluble sugar, and cellulose contents, were higher than those of the control, while the content of nitrite and lignin was lower than that of the control. Hence, EBR pretreatment was able to enhance the antioxidant capacity of wucai, maintain normal leaf color and shape during storage, and delay the decline of nutritional properties; therefore, EBR pretreatment has potential commercial value for prolonging the market life of wucai.

## Graphical abstract

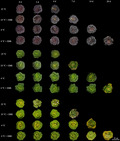

## 1 INTRODUCTION

Wucai (*Brassica campestris* L. ssp. *chinensis* var. *rosularis* Tsen et Lee), a subspecies of nonheading Chinese cabbage, is widely grown in the Yangtze–Huai River Basin (Yuan et al., 2019). This crop is very appreciated by consumers for its taste and high nutritional value (Yuan et al., 2016). It is adapted to grow well in cold weather and ripens around the time of the Spring Festival in China. Wucai varieties of different colors greatly satisfy the needs of the customers. According to the variety, the color of wucai leaves varies and can be green, yellow‐green, or purple. Recently, the wucai with purple leaves has attracted increasing attention as a potential source of nutraceuticals owing to their high anthocyanin level and microelements, which are beneficial to human health.

However, wucai is prone to dehydration and yellowing during storage, as are other leafy green vegetables, due to its relatively high respiration rate, high water content, and pigment degradation after harvesting (Viškelienė et al., 2017). Meanwhile, the loss and degradation of the quality of postharvest green vegetables is manifested by yellowing due to chlorophyll (Chl) degradation, wilting and loss of textural properties, and decay caused by pathology (Ambuko et al., 2017). Therefore, it is of great significance to study the maintenance of nutrients and ways in which to extend the shelf life of wucai during the postharvest period.

To enhance the storability of leafy vegetables and lengthen their shelf life, many physical and chemical methods are widely used in the postharvest leafy vegetable industry. One of the most common ways to maintain postharvest nutritional quality of leafy vegetables is to use low‐temperature storage. According to Porter et al. (2003), low‐temperature storage reduces respiration rate, ethylene production and sensitivity, moisture loss, and the growth of pathogens. However, some leafy vegetables may suffer from chilling injury during low‐temperature storage. Previous study reported that the main limiting factors of pak choy on shelf life were yellowing at 10°C and wilting at 2°C, and the pak choy could be stored at 10°C for 8 days and 2°C for about and 27 days (Able et al., 2005). Thus, many chemical preservation methods, such as cholesterol (Gu et al., 2019), 1‐methylcyclopropene (Able et al., 2002), and salicylates (Martinez‐Espla et al., 2018), combined with low temperature have been applied to maintain the nutritional quality of vegetables and prolong the postharvest life. Nevertheless, researchers still need more comprehensive tests to determine whether such chemicals pose a potential threat to consumer health. Therefore, it is extremely necessary to explore a safe and efficient way to prolong the shelf life of postharvest vegetables.

Brassinosteroids (BRs) are a class of growth‐promoting steroid hormones, which widely exist in plants and regulate many aspects of physiological responses necessary for nutrition and reproductive development (Clouse, 2011). BRs are considered to be the sixth class of plant hormones (Peres et al., 2019). In recent years, EBR, one type of BR, has been considered to play pivotal roles in tolerance to biotic and abiotic stress. A growing number of studies have reported that EBR played an important role in extending the shelf life of fruits and vegetables (Gao et al., 2016); for example, it was shown to significantly delay jujube fruit senescence and maintain fruit quality by reducing ethylene production and inhibiting respiration rate (Zhu et al., 2010). EBR treatment significantly reduced the production of endogenous ethylene in broccoli and delayed yellowing (Cai et al., 2019). However, the effect of EBR on postharvest storage of wucai has not been reported. Overall, it is of great significance to evaluate the effect of EBR on the senescence process of wucai, which could provide a better method for wucai storage.

The purpose of this study was to evaluate the effects of EBR on the storage and nutritional quality of wucai (three varieties, each having a different leaf color). In this study, we further investigated the effect of EBR pretreatment on weight loss, electrolyte leakage, pigment content, nutritional quality, antioxidants, and the AsA‐GSH cycle during postharvest storage of wucai.

## 2 MATERIALS AND METHODS

### 2.1 Plant materials and treatments

Three genotypes of wucai with different colored leaves, PW‐13 (purple leaf), GW16‐28 (green leaf), and YW15‐8 (yellow leaf), were selected as representative varieties for the present study. The seeds of the three experimental varieties were from the Vegetable Genetics and Breeding Laboratory of Anhui Agricultural University (Hefei, China). The three wucai seeds were planted in the glasshouse of Anhui Agricultural University. Three genotypes wucai reached the commercial harvest period after 56 days of growth.

A preliminary experiment to evaluate the visual quality of wucai pretreated with different concentrations (0, 0.05, 0.1, 0.15, and 0.2 μM) of EBR was completed (Figures [Link], [Link], [Link]). Consequently, 0.1 μM EBR, which was the optimum concentration, was selected for the present study. EBR was sprayed on foliage twice at 48 and 24 hr before harvesting. Plants that were similar in size and had no mechanical damage were selected as samples. And the samples were washed with water and wiped dry before packaging. Each of the three sample types was divided randomly into four groups, packed as whole foliage into polythene plastic bags (0.1 mm), and stored at 25°C and 4°C with 85% relative humidity. The treatments were as follows.

- 0 μM EBR pretreatments, stored at 25°C (Cont);
- 0.1 μM EBR pretreatments, stored at 25°C (Cont + EBR);
- 0 μM EBR pretreatments, stored at 4°C (LT);
- 0.1 μM EBR pretreatments, stored at 4°C (LT + EBR).

Each treatment has three replicate samples, and three subsamples were taken for each biological replicate. According to storage time, samples were collected at 0, 2, 4, 7, and 14 days, and physiological and biochemical measurements were taken. Analysis of free amino acid content of three different cultivars in four treatments was conducted on the fourth day. Total RNA was extracted from leaf of GW16‐28 at 0 and 4 days under 25°C and 0, 4, and 7 days under 4°C.

### 2.2 Measurements of weight loss rate, color values, and pigment content

The weight loss rate was defined as the ratio of final sample weights to initial sample weights (Meng et al., 2012). A colorimeter (CR‐400‐C, Konica Minolta Sensing Americas, Inc.) was used to evaluate the chromatic characteristics of plant leaves as color parameters *L* ^*^, *a* ^*^, and *b* ^*^ (Lu et al., 2010). Total chromatism value (Δ*E*) using the following equation:

(1) $$\Delta E=\sqrt{{\left(L_{0}‐L*\right)}^{2}+{\left(a_{0}‐a*\right)}^{2}+{\left(b_{0}‐b*\right)}^{2}}$$

The change in Δ*E* value indicates how fast the leaf color of the plant changes. The pigment content was measured by the method of Arnon (1949) with slight modification. Fresh leaves (0.2 g) were extracted for 26 hr in the dark in 25 ml extract buffer (acetone:ethanol:water = 4.5:4.5:1). The absorbance was determined with a UV‐vis spectrophotometer (TU1950, PERSEE) at 665, 649, and 470 nm.

### 2.3 Measurements of relative electrical conductivity (EC) and malondialdehyde (MDA) content

EC was measured according to the method described by Baziramakenga et al. (1995) with some modifications. The leaf disks (7.5 mm in diameter) were placed in 20 ml deionized water for 30 min. The conductivity of the leaf soak water (*L*_1_) and deionized water (*L*_0_) was measured by an Orion STARA112 conductivity meter (Thermo Scientific). The test tubes were placed in boiling water for 10 min and cooled to room temperature. Also, the conductivity *L*_2_ was measured. The EC was calculated according to the formula:

(2) $$\text{EC}=\left(L_{1}‐L_{0}\right)/\left(L_{2}‐L_{0}\right)\times 100\%$$

For the measurement of MDA content, fresh leaves (0.2 g) were added to 1.6 ml trichloroacetic acid (10%, v/v) and centrifuged at 12,000 *g* for 10 min. The supernatant (1.5 ml) with 1.5 ml thiobarbituric acid (0.67%) was boiled for 15 min and centrifuged at 10,000 *g* for 10 min. The absorbance was measured at 450, 532, and 600 nm (Liu et al., 2006).

### 2.4 Measurements of hydrogen peroxide content (H_2_O_2_) and total antioxidant capacity (T‐AOC)

H_2_O_2_ content and T‐AOC were measured using a Solarbio reagent kit (Cat #BC3595 and 1315, Beijing Solarbio Science & Technology Co., Ltd.).

### 2.5 Measurement of total soluble sugar content

The content of total soluble sugar was each measured by the anthrone colorimetric method (Laurentin & Edwards, 2003). Dried samples (50 mg) were mixed with 4 ml alcohol (80%, v/v) and shaken at 80°C for 30 min. The residue was extracted with 80% alcohol. The extract (20 μl) was added to 480 μl water and 2.5 ml anthrone (72%, v/v), then boiled at 90°C for 15 min. The absorbance was determined with a UV‐vis spectrophotometer (TU1950, PERSEE) at 620 nm.

### 2.6 Analysis of free amino acid content

Total free amino acid content was quantified using the method reported by Lee and Takahashi (1966). Fresh leaves (0.5 g) were triturated with 5 ml acetic acid (10%, v/v) and then diluted to 100 ml. One milliliter of the filtrate was combined with 3 ml ninhydrin, 1 ml acetic acid–sodium acetate buffer (pH = 5.4), and 0.1 ml 0.1% AsA, and boiled for 15 min. The absorbance at 570 nm was recorded. Each free amino acid was quantitatively evaluated using an amino acid analyzer (High‐speed Amino Acid Analyzer L‐8900, Hitachi High‐Technologies Corporation).

### 2.7 Measurements of cellulose, lignin, and nitrite contents

Cellulose content was determined using the anthrone method (Viles & Silverman, 1949). Dried samples (100 mg) were digested with sulfuric acid. Two milliliters of the filtrate was combined with 0.5 ml of 2% anthrone solution and 5 ml of sulfuric acid. The absorbance was measured at 620 nm. Lignin content was measured with a Solarbio reagent kit. The nitrite content was measured with the official method GB 5009.33‐2016 of the National Standard of the People's Republic of China.

### 2.8 Enzyme activity assays in the AsA‐GSH cycle

The ascorbate peroxidase (APX) activity was assayed as described by Nakano and Asada (1981). Assay of glutathione reductase (GR) activity was carried out according to Cakmak and Marschner (1992). The dehydroascorbate reductase (DHAR) and monodehydroascorbate reductase (MDHAR) activities were assayed according to Dalton et al. (1986).

### 2.9 Measurement of intermediate metabolite content in the AsA‐GSH pool

The content of GSH and oxidized glutathione (GSSG) was measured according to the Solarbio reagent kit. The oxalic acid method was used for the determination of AsA levels in wucai leaves (Zhou et al., 2017). The reaction solution was used to assay AsA and dehydroascorbate (DHA) content by high‐performance liquid chromatography (Thermo Fisher Scientific).

### 2.10 Quantitative RT‐PCR analysis for AsA metabolism‐related genes

Total RNA was extracted from each sample using the Plant RNA Extraction Kit (Takara Biomedical Technology Co.). After determining the integrity and concentration of RNA, the DNase‐treated RNA (1 mg) was reverse transcribed to cDNA using the PrimeScript™ RT Reagent Kit (TaKaRa). Quantitative real‐time PCR (qRT‐PCR) was then performed with the SYBR^®^ Premix Ex Taq™ II Kit (TaKaRa). The *BnaActin* gene was used as the control gene. The gene‐specific primers were designed using Primer software version 5.0 (Premier Biosoft International) for the qRT‐PCR (Table S1) of the genes coding for APX, AAO, DHAR, and MDHAR. Three biological repeats for each sample and three technical replicates for each gene were performed, and the relative expression levels were calculated as 2^−ΔΔCt^.

### 2.11 Statistical analysis

Experiments were performed using a completely randomized design. All data are reported as mean ± standard deviation, and three biological replicates were performed. All data were statistically analyzed using one‐way analysis of variance (ANOVA) with SPSS 22.0 (SPSS Institute Inc., Chicago, IL, USA), and means were separated using Tukey's test at a significance level of *p* < .05. GraphPad Prism software (GraphPad Software, San Diego, CA, USA) was used for diagraph analysis.

## 3 RESULTS AND DISCUSSION

### 3.1 Effects of exogenous EBR on the shelf life of wucai

**FIGURE 1:**
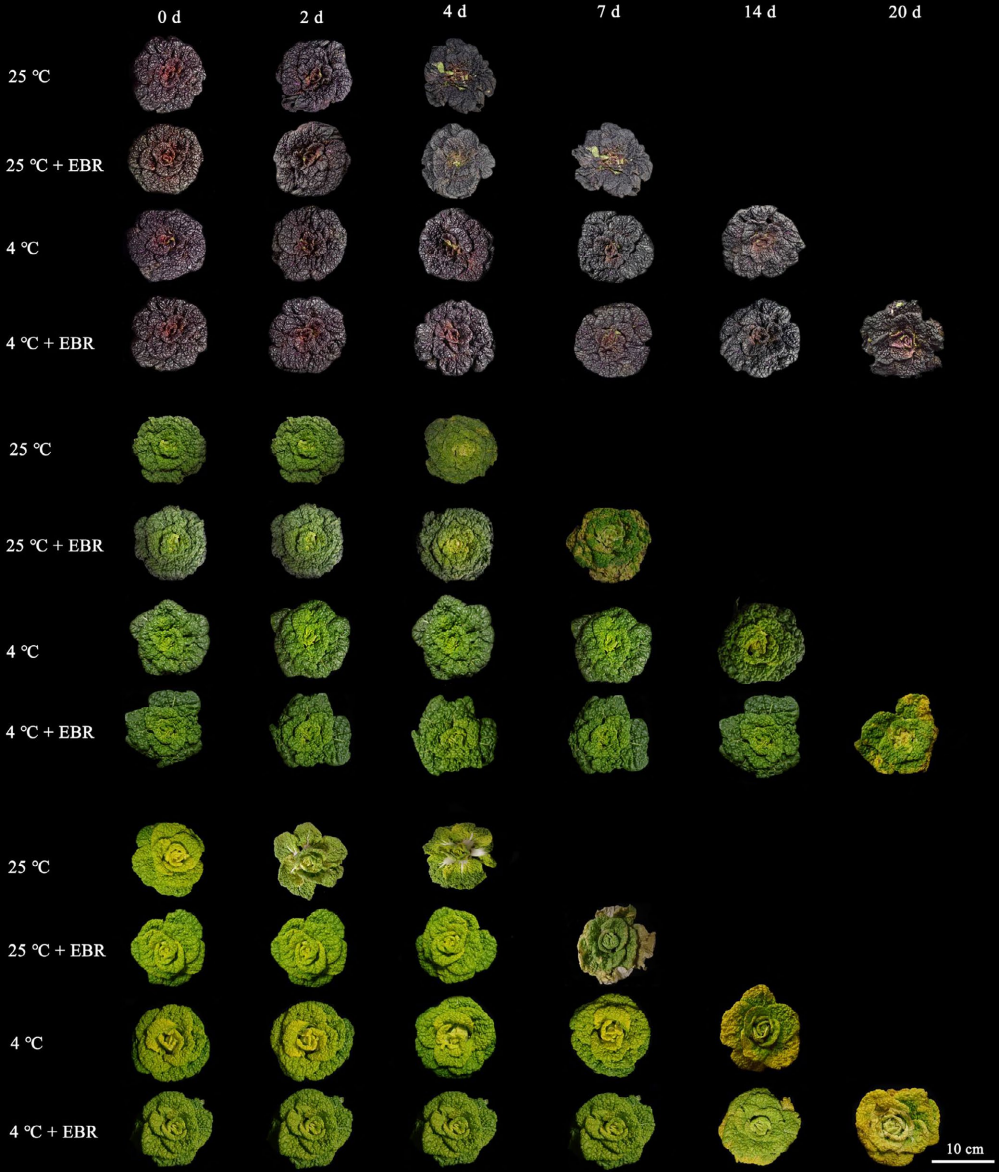
Postharvest storage appearance of three different colored varieties of wucai (PW‐13, GW16‐28, and YW15‐8) under four treatments. The scale bar is 10 cm

There was a considerable difference in shelf life in four treatment groups. Figure 1 shows the morphological changes in the three different colored varieties of wucai as storage time progresses. Compared to 25°C, the shelf life of all treatments was significantly extended at 4°C. Also, the exogenous EBR application further delayed the senescence of all groups. At 4°C, all types of wucai presented noticeable wilt after 14 days, whereas the EBR pretreatment delayed the senescence, and leaves became yellow after 20 days. However, the sample could be stored for only 7 days at 4°C without EBR application, and the plants had lost water and wilted after 4 days at 25°C. This was consistent with previous research on kiwifruit (Wang, Lu, et al., 2020) and broccoli (Cai et al., 2019).

### 3.2 Color values, pigment content, and weight loss analysis

**FIGURE 2:**
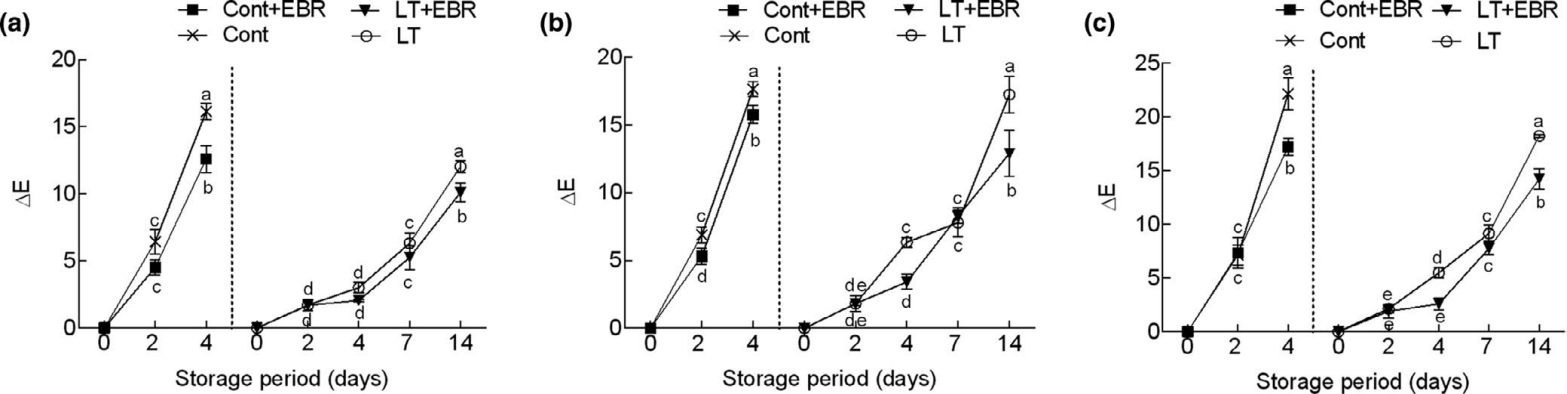
Influence of EBR pretreatment on the total chromatism value (Δ*E*) in PW‐13 (a), GW16‐28 (b), and YW15‐8 (c) during the storage period at different temperatures. Each data point represents the mean ± standard deviation from three separate experiments. Different letters within a column indicate significant differences at *p* < .05

**FIGURE 3:**
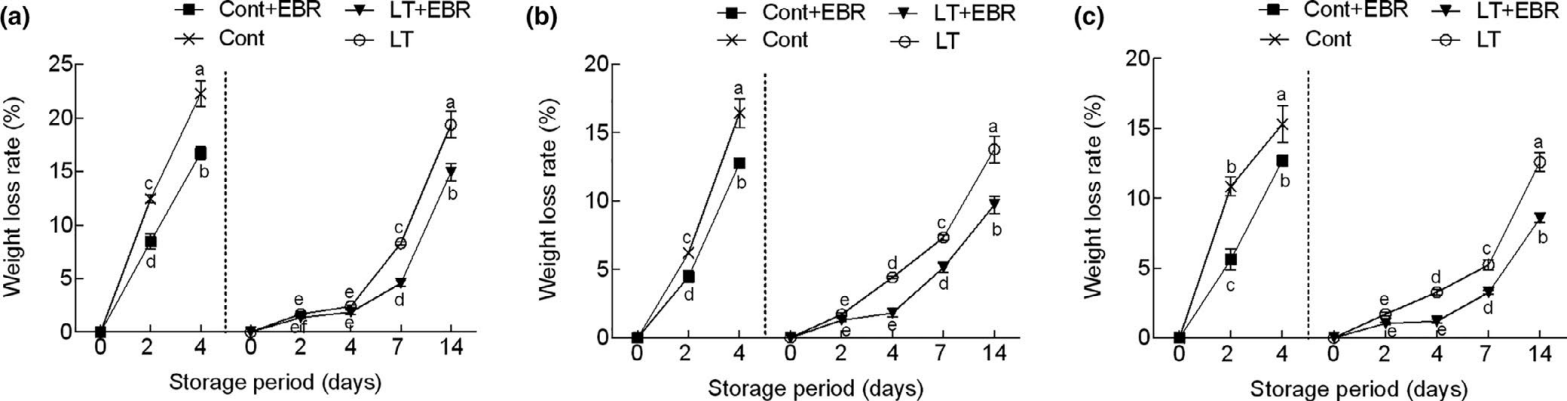
Influence of EBR pretreatment on the weight loss rate in PW‐13 (a), GW16‐28 (b), and YW15‐8 (c) during the storage period at different temperatures. Each data point represents the mean ± standard deviation from three separate experiments. Different letters within a column indicate significant differences at *p* < .05

Water loss and pigment degradation are major factors that reduce postharvest quality and limit the shelf life of leafy vegetables (Mu et al., 2020). In this study, the Δ*E* value of the three wucai varieties continuously increased over the storage period (Figure 2). For both temperatures, the Δ*E* value in the control gradually rose during the entire storage period, while EBR pretreatment significantly (*p* < .05) reduced this change. When GW16‐28 was stored at 4°C for 14 days, the Δ*E* value reached 17.24%, but was only 12.90% after EBR was sprayed. Similarly, a general trend of a decrease in Chl content was observed as the storage time increased in all treatments (Figures S4 and S5). Conversely, the carotenoid (Car) content increased continuously (Figure S6). Compared to 25°C, the Chl content decreased more slowly at 4°C. Also, the EBR pretreatment significantly (*p* < .05) inhibited Chl degradation throughout storage. Both the control and EBR‐pretreated samples showed an increase in weight loss rate with storage duration, but EBR pretreatment significantly (*p* < .05) delayed this process, especially within 4 days after EBR pretreatment (Figure 3). During the 4°C storage of GW16‐28, the weight loss rate increased under the LT group, reaching 13.77% at 14 days, and EBR pretreatment significantly (*p* < .05) inhibited this change and reached 9.70%.

The color values, Chl content, and weight loss rate are the primary visual qualities of greatest concern in the preservation of wucai, which directly influence its commercial value. The current results show that EBR pretreatment can significantly inhibit the water loss of wucai and pigment degradation during storage. Also, with EBR pretreatment, the three varieties of wucai changed more slowly than the control group did during the entire storage period, resulting in an extended shelf life of the EBR‐pretreated samples. It suggests that EBR may be an effective method for delaying senescence and extending shelf life of wucai, especially in low‐temperature environments. Similar effects were found in daylily flower buds (Yao et al., 2017), broccoli (Cai et al., 2019), jujube (Zhu et al., 2010), and kiwifruit (Lu et al., 2019).

### 3.3 Changes in EC, MDA, H_2_O_2_ content, and T‐AOC

**FIGURE 4:**
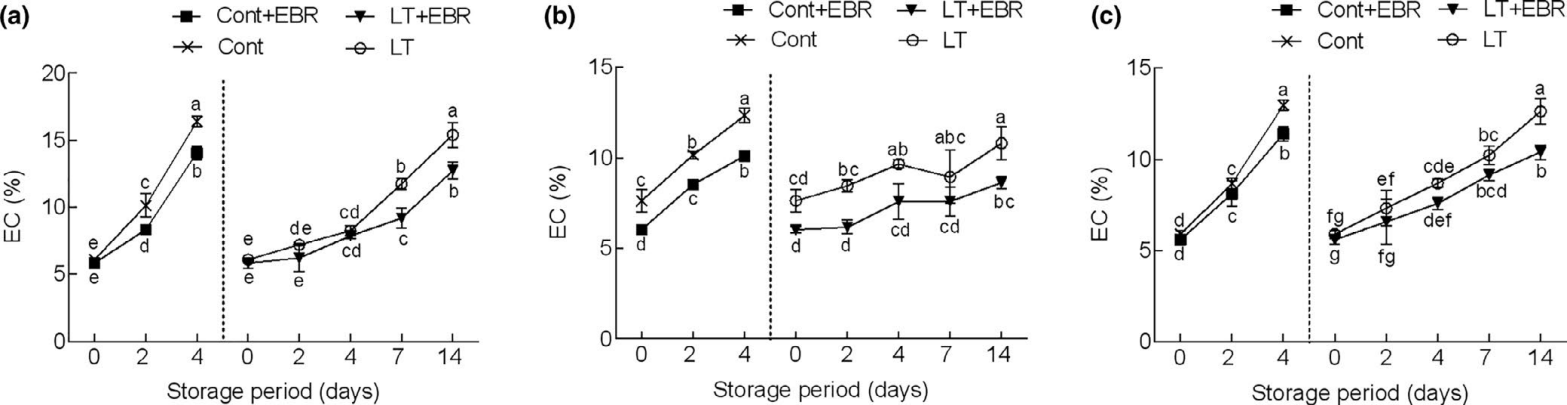
Influence of EBR pretreatment on relative electrical conductivity (EC) PW‐13 (a), GW16‐28 (b), and YW15‐8 (c) during the storage period at different temperatures. Each data point represents the mean ± standard deviation from three separate experiments. Different letters within a column indicate significant differences at *p* < .05

**FIGURE 5:**
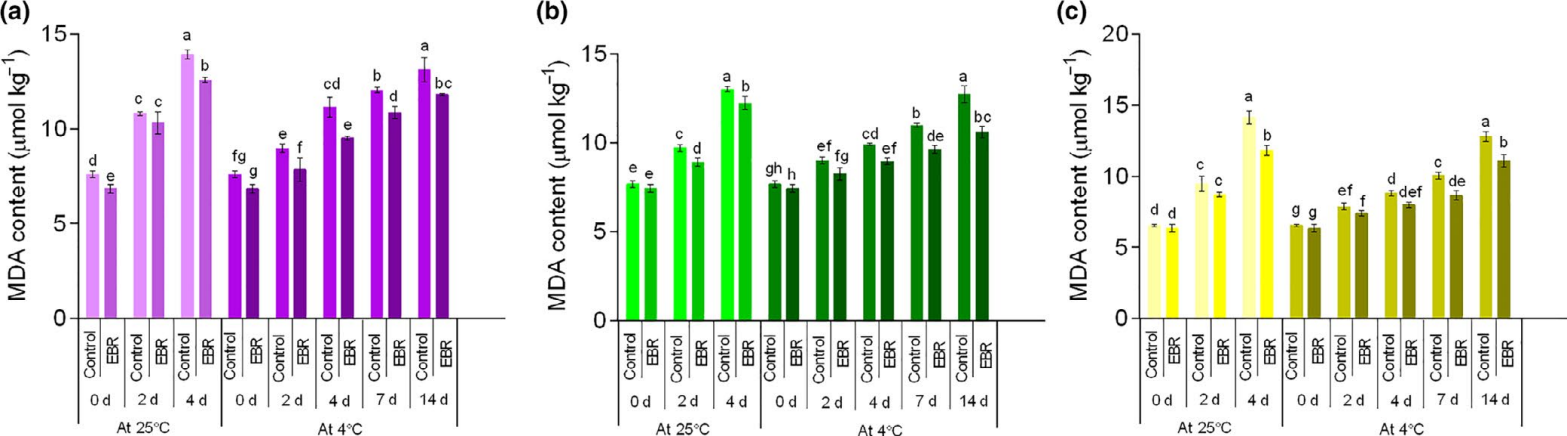
Changes in the content of malondialdehyde (MDA) in PW‐13 (a), GW16‐28 (b), and YW15‐8 (c) during the storage period under four treatments. Each data point represents the mean ± standard deviation from three separate experiments. Different letters within a column indicate significant differences at *p* < .05

The accumulation of reactive oxygen species (ROS) is closely related to plant senescence and is an inevitable result of aerobic metabolism during postharvest storage, which leads to oxidative damage and lipid peroxidation of plant cell membranes (Nejadsadeghi et al., 2015). Both control and EBR‐pretreated wucai showed an almost linear increase in EC during storage, but the EBR pretreatment prevented this increase (Figure 4). At the end of storage at 4°C, the EC of GW16‐28 pretreated with EBR was 8.63%, which was about 1.25 times lower than that of the LT group. As the storage period increased, the content of MDA in the three types of samples slowly increased. Also, MDA content in EBR‐pretreated wucai was lower (*p* < .05) than that of the control during the entire storage period (Figure 5). For YW15‐8, an approximate 16.57% decrease in MDA was recorded after 4 days of 25°C storage compared with that of the control.

**FIGURE 6:**
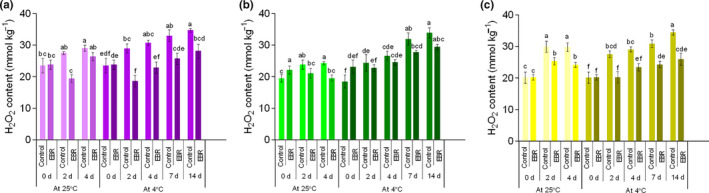
Changes in the content of hydrogen peroxide (H_2_O_2_) in PW‐13 (a), GW16‐28 (b), and YW15‐8 (c) during the storage period under four treatments. Each data point represents the mean ± standard deviation from three separate experiments. Different letters within a column indicate significant differences at *p* < .05

H_2_O_2_ content and T‐AOC of wucai presented an overall rising trend during the whole storage period. Nevertheless, EBR pretreatment materially (*p* < .05) inhibited the rise of the H_2_O_2_ content (Figure 6) and T‐AOC (Figure S7). Low temperature also had a certain inhibitory effect on the rise. In PW‐13, the H_2_O_2_ content of the LT group was 28.34% higher than that of EBR‐pretreated sample when stored at 4°C for 7 days. Under the two temperature conditions, the T‐AOC of the EBR pretreatment in all materials was significantly (*p* < .05) higher than that of the control at the end of storage. In PW‐3, the T‐AOC after the EBR pretreatment was 1.18 times that of the LT group when stored at 4°C for 14 days. These results are consistent with those of a previous study on table grapes (Liu, Xi, et al., 2016). The T‐AOC gradually increased throughout the storage period, indicating that the antioxidants played a significant role in scavenging ROS. EBR pretreatment significantly enhanced the antioxidant capacity of the plants, which was consistent with findings of EBR treatment of pak choy (Al Ubeed et al., 2019).

### 3.4 Analysis of total soluble sugar and free amino acids

**FIGURE 7:**
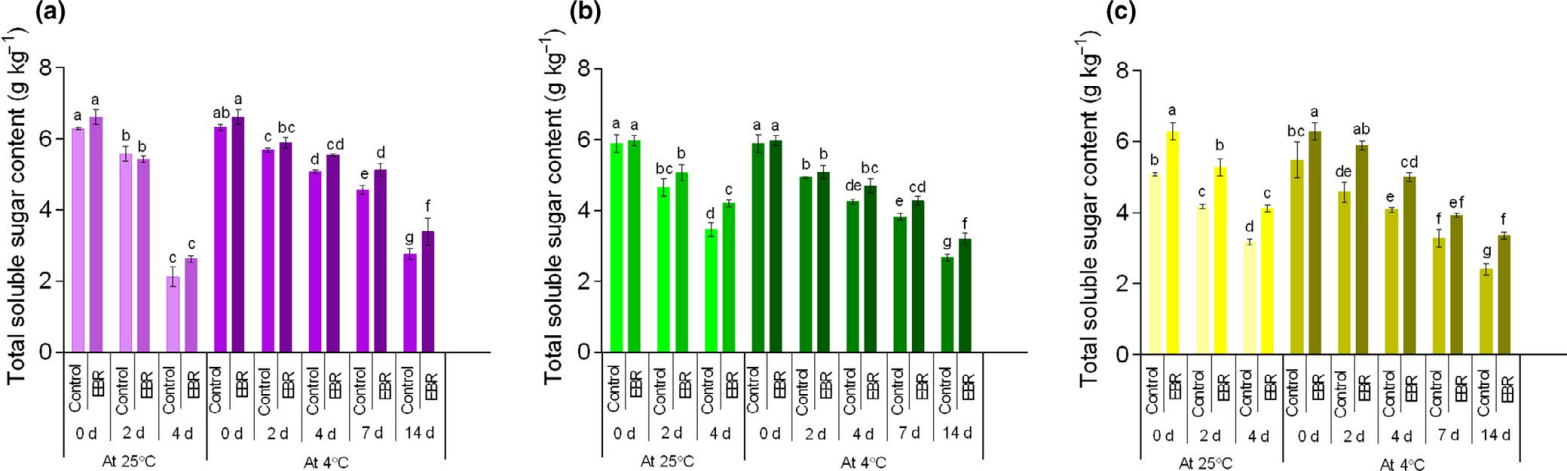
Changes in total soluble sugar content in PW‐13 (a), GW16‐28 (b), and YW15‐8 (c) during the storage period under four treatments. Each data point represents the mean ± standard deviation from three separate experiments. Different letters within a column indicate significant differences at *p* < .05

The total soluble sugar content in the control decreased rapidly, especially at 25°C (Figure 7). Nevertheless, this decrease was markedly (*p* < .05) delayed by EBR. By the end of the 4°C storage in YW15‐8, total soluble sugar content in the LT group decreased rapidly; it decreased by 56.01%, while it decreased by 46.56% under the EBR pretreatment. Soluble carbohydrates play an important role in plants under stress (Wang, Lv, et al., 2020). Previous research reports that sugar can be used as a ROS scavenger to enhance tolerance in plants (Lu et al., 2019). An increase in sucrose content helped improve the cold tolerance of nectarine fruits (Zhao et al., 2019).

**FIGURE 8:**
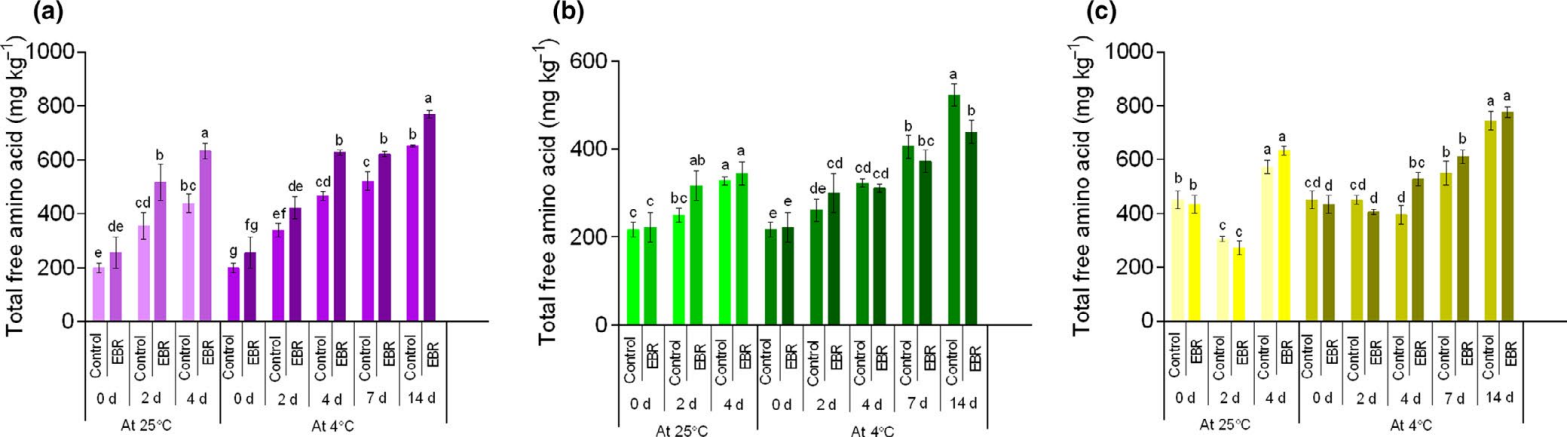
Changes in the content of total free amino acid in PW‐13 (a), GW16‐28 (b), and YW15‐8 (c) during the storage period under four treatments. Each data point represents the mean ± standard deviation from three separate experiments. Different letters within a column indicate significant differences at *p* < .05

Free amino acids play an important role in the detoxification of toxic elements. The results show that plants exposed to toxic elements accumulate histidine, proline, and glycine, which participate in the regulation of ion transport and exert osmotic adjustment function (Okunev, 2019). In this study, the content of the total free amino acid of the three experimental materials increased gradually with storage time. Also, the EBR pretreatment could promote the increase in the total free amino acid content. As for PW‐13 stored at 4°C for 14 days, the total free amino acid content of the EBR pretreatment increased 1.96 times compared with 0 day (Figure 8a). Eighteen individual free amino acids were quantified in the three types of wucai that stored for 4 days (Tables S2‐S4). In the different treatments, the contents of alanine, arginine, proline, and threonine were the top four free amino acids. It is worth mentioning that the proline content of EBR‐pretreated samples was significantly higher than that of the LT group. Proline metabolism plays an important role in increasing cell cellular osmolarity, stabilizing membrane and subcellular structures, and protecting cells from oxidative damage under stress (Liu, Li, et al., 2016). Likewise, there is reportedly an increase in the content of proline with EBR pretreatment in bamboo shoot (Kaur et al., 2011) and jujube fruit (Zhu et al., 2010).

### 3.5 Analysis of cellulose, lignin, and nitrite

**FIGURE 9:**
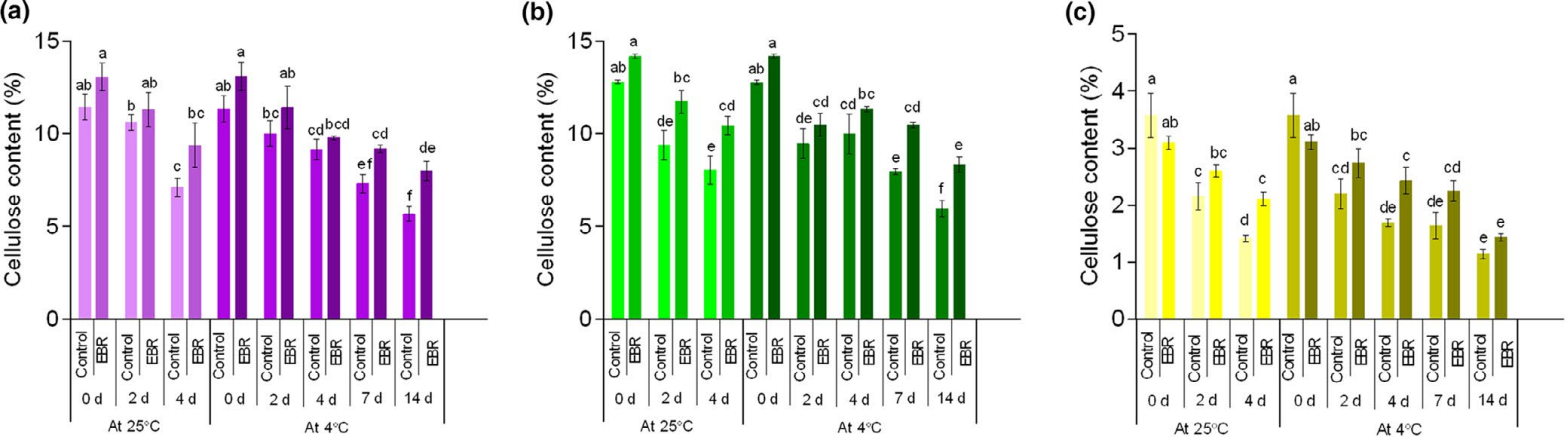
Changes in cellulose content in PW‐13 (a), GW16‐28 (b), and YW15‐8 (c) during the storage period under four treatments. Each data point represents the mean ± standard deviation from three separate experiments. Different letters within a column indicate significant differences at *p* < .05

For cellulose, the content of three varieties of wucai continued to decrease throughout the storage period, while EBR pretreatment significantly (*p* < .05) inhibited its decline (Figure 9). To a certain extent, the low‐temperature environment also suppressed its decline. At 4°C storage, the cellulose content of GW16‐28 in the LT group decreased continuously and decreased by 53.28% at 14 days of storage, while the EBR pretreatment significantly (*p* < .05) inhibited this change, which only decreased by 41.18% at the same time of storage. As for lignin and nitrite, the EBR pretreatment significantly slowed down the increase in these two components as compared to control (Figures S8 and S9). Also compared to 25°C, the level of lignin content and nitrite content rose more slowly. Both lignin content and nitrite content in the 25°C treated group were noticeably higher than that in the 4°C treated group during the entire storage period. Cellulose as a structural element of cell wall can also be used as a form of energy storage (Yu et al., 2019). Decomposition of cellulose and lignin causes the softening of plant leaves. The postharvest structural deterioration of leafy vegetables is related to changes in the structure of the cell wall, mainly due to the degradation of lignin, hemicellulose, and cellulose caused by the action of cell wall hydrolases (Shi et al., 2019).

### 3.6 Related enzymatic activities of AsA‐GSH cycle

In order to combat ROS accumulation, plants have developed complex systems including antioxidant enzymes and metabolites. The AsA‐GSH cycle is an important system against H_2_O_2_ generation (Palma et al., 2006). It has been reported that increasing the activity levels of the enzymatic components (MDHAR, DHAR, and GR) contributes to the regeneration of the nonenzymatic components (AsA and GSH) to maintain the maintenance of mitochondrial redox homeostasis during tomato fruit ripening (Lopez‐Vidal et al., 2016). As shown in Figure S10, the activity of APX was significantly (*p* < .05) enhanced by EBR pretreatment. At 4°C, the activity of APX in GW16‐28 peaked on day 4 with the EBR pretreatment, which was 2.74 times of that at 0 day. Similarly, the MDHAR activity in all samples stored at 4°C reached a peak at 4 days (Figure S11). In GW16‐28, MDHAR activity in the EBR pretreatment and the LT group reached a peak level at 4 days, which was increased by 67.14% and 50.82%, respectively, over that stored at 0 day. For DHAR activity, there was a continuous increase at 4°C in the entire storage period (Figure S12). EBR pretreatment clearly promoted the increase in DHAR activity. When YW15‐8 was stored at 4°C for 14 days, the DHAR activity of the EBR pretreatment was 1.32 times that of the LT group. In addition, the GR activity in wucai was significantly (*p* < .05) increased by the EBR pretreatment (Figure S13). Also, the GR activity in the leaves pretreated with EBR showed an increasing trend, except for PW‐13.

### 3.7 Intermediate metabolites of the AsA‐GSH pool

The results showed a significant (*p* < .05) difference in AsA content between the EBR‐pretreated materials and control materials (Table 1). EBR caused a significant (*p* < .05) increase in AsA, GSH, and GSSG contents (Table 2). For PW‐13 stored at 25°C for 4 days, the level of AsA pretreated by EBR was 1.13 times that of the control. Moreover, among the three varieties of wucai, the AsA content of PW‐13 was much higher than that of the other varieties. At 4°C, the GSH content of YW15‐8 pretreated with EBR increased 1.31‐fold compared with the initial level. In addition, the ratio of AsA/DHA and GSH/GSSG markedly increased in all plants that were pretreated with EBR. This indicates that exogenous EBR can improve the antioxidant system activity in wucai plants and promote the AsA‐GSH cycle to maintain its normal state, which is consistent with the findings of previous studies in eggplant (Wu et al., 2014), pepper (Wang et al., 2012), and grapevine (Chen et al., 2019).

**TABLE 1:** Effects of different treatments for three varieties of wucai on the contents of AsA, DHA, and AsA/DHA ratios at 4 days

Treatments	AsA (mg/kg)	DHA (mg/kg)	AsA/DHA ratio
PW‐13 25°C	988.64 ± 6.23 ^d^	136.31 ± 6.67 ^b^	7.26 ± 0.36 ^b^
25°C + EBR	1,116.06 ± 8.47 ^c^	141.34 ± 9.69 ^b^	7.92 ± 0.59 ^b^
4°C	1,196.22 ± 7.07 ^b^	108.36 ± 11.32 ^c^	11.12 ± 1.16 ^a^
4°C + EBR	1,242.29 ± 5.07 ^a^	186.63 ± 1.35 ^a^	6.65 ± 0.07 ^b^
GW16‐28 25°C	341.49 ± 4.16 ^d^	63.06 ± 6.55 ^a^	5.45 ± 0.56 ^c^
25°C + EBR	457.05 ± 4.03 ^a^	45.02 ± 4.27 ^b^	10.21 ± 0.94 ^a^
4°C	360.95 ± 2.32 ^c^	46.54 ± 2.27 ^b^	7.76 ± 0.42 ^b^
4°C + EBR	440.25 ± 1.16 ^b^	65.24 ± 7.66 ^a^	6.81 ± 0.86 ^bc^
YW15‐8 25°C	345.49 ± 6.15 ^c^	41.63 ± 2.35 ^bc^	8.31 ± 0.57 ^b^
25°C + EBR	403.18 ± 9.99 ^a^	30.75 ± 6.09 ^c^	13.54 ± 3.32 ^a^
4°C	381.81 ± 4.16 ^b^	51.04 ± 1.11 ^ab^	7.48 ± 0.22 ^b^
4°C + EBR	398.58 ± 5.33 ^a^	61.82 ± 9.31 ^a^	6.54 ± 0.93 ^b^

Note

Each data point represents the mean ± standard deviation from three separate experiments. Different letters within a column indicate significant differences at *p* < .05.

**TABLE 2:** Effects of different treatments for three varieties of wucai on the contents of GSH, GSSG, and GSH/GSSG ratio at 4 days

Treatments	GSH (mg/kg)	GSSG (mg/kg)	GSH/GSSG ratio
PW‐13 25°C	157.29 ± 11.31 ^bc^	113.57 ± 2.25 ^ab^	1.38 ± 0.08 ^c^
25°C + EBR	169.57 ± 3.60 ^ab^	122.06 ± 4.05 ^a^	1.39 ± 0.07 ^b^
4°C	147.64 ± 10.19 ^c^	105.51 ± 2.83 ^b^	1.40 ± 0.09 ^b^
4°C + EBR	185.36 ± 22.84 ^a^	112.77 ± 6.96 ^ab^	1.65 ± 0.17 ^a^
GW16‐28 25°C	51.59 ± 2.31 ^c^	35.13 ± 2.95 ^b^	1.47 ± 0.09 ^c^
25°C + EBR	82.71 ± 13.21 ^bc^	45.94 ± 6.97 ^a^	1.80 ± 0.11 ^bc^
4°C	101.13 ± 17.15 ^b^	35.85 ± 3.87 ^b^	2.86 ± 0.65 ^ab^
4°C + EBR	146.58 ± 24.88 ^a^	45.22 ± 6.79 ^a^	3.29 ± 0.64 ^a^
YW15‐8 25°C	104.64 ± 3.60 ^c^	45.62 ± 2.95 ^c^	2.29 ± 0.27 ^b^
25°C + EBR	122.19 ± 12.36 ^b^	51.98 ± 1.93 ^b^	2.35 ± 0.16 ^ab^
4°C	112.98 ± 6.48 ^bc^	52.42 ± 1.12 ^b^	2.15 ± 0.10 ^c^
4°C + EBR	143.25 ± 12.70 ^a^	59.63 ± 4.02 ^a^	2.40 ± 0.10 ^a^

Note

Each data point represents the mean ± standard deviation from three separate experiments. Different letters within a column indicate significant differences at *p* < .05.

### 3.8 Expression of AsA metabolism‐related genes

**FIGURE 10:**
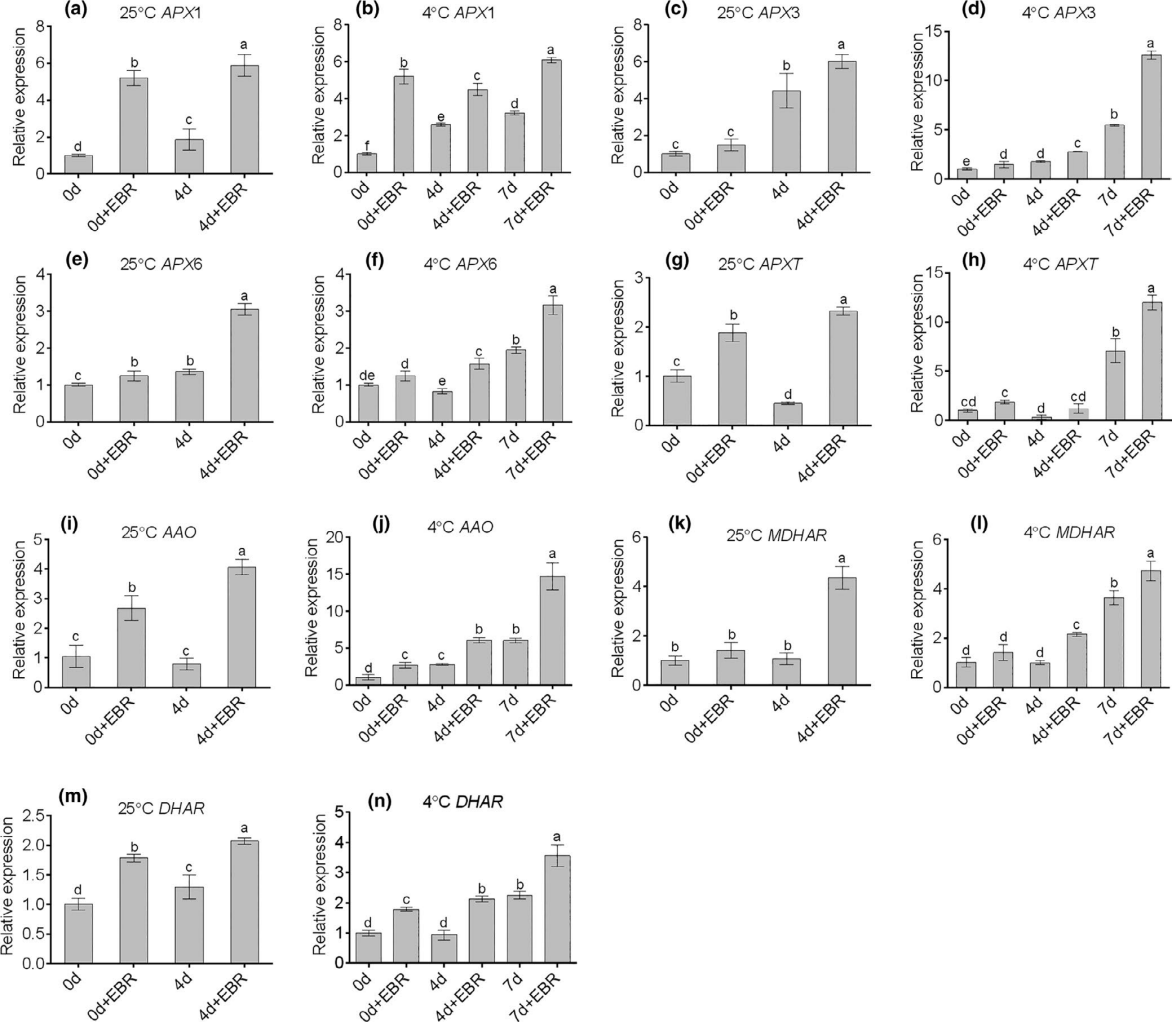
Relative expressions of the AsA metabolism‐related genes during the storage period at different temperatures. Total RNA was extracted from leaf of GW16‐28 at 0 and 4 days under 25°C and 0, 4, and 7 days under 4°C. The *BnaActin* gene was used as the internal control to calculate the relative expression level. Data shown here are mean ± standard deviation of three biological replicates. Different letters within a column indicate significant differences at *p* < .05

To analyze the possible molecular mechanisms of EBR‐induced antioxidant capacity, seven AsA metabolism‐related genes were assayed. As shown in Figure 10, AsA metabolism‐related genes were significantly induced in EBR‐pretreated leaves during the entire storage period. Overall, the expression levels of *APX1*, *APX3*, *APX6*, *APXT*, *AAO*, *MDHAR*, and *DHAR* genes increased with EBR pretreatment, and at 4°C, the expression levels increased significantly (*p* < .05) for samples pretreated with EBR for 7 days. In agreement, the enzymatic activity of APX, MDHAR, and DHAR also increased, and this result was basically consistent with the gene expression level. The AsA metabolism pathway has been extensively studied (Cui et al., 2017; Wu et al., 2017). The present research examined several key genes related to AsA metabolism coding for AAO, APX, MDHAR, and DHAR in wucai and found that the expression of *APX1*, *APX3, APX6, APXT, AAO*, *MDHAR*, and *DHAR* was significantly upregulated with EBR pretreatment. A similar report in mini Chinese cabbage (Hu et al., 2019) showed that nitrate treatments upregulated the relative expression levels of the *GR*, *MDHAR1*, *APXT*, *DHAR2*, and *AAO* genes, thereby improving the resistance to stress.

## 4 CONCLUSIONS

In summary, after 4 days at room temperature, wucai had rotted and thus lost its commercial value. However, at 4°C, its shelf life was effectively extended, and the loss of nutritional quality was alleviated. EBR, as a safe and environmentally friendly compound, has great benefits in maintaining plant morphology and nutritional quality. EBR pretreatment effectively reduced the decrease in water loss and pigment degradation to maintain normal morphology of wucai. It provides a positive effect for wucai to enhance antioxidant capacity by decreasing of H_2_O_2_ content and increasing T‐AOC and AsA‐GSH cycle‐related enzyme activity. Meanwhile, EBR pretreatment showed positive effects on nutritional quality parameters. EBR pretreatment delayed the decrease in total soluble sugar, sucrose, starch, and cellulose in the plant, promoted the increase in free amino acid and fructose contents, and inhibited the increase in lignin and nitrite contents. The results show that EBR pretreatment could delay the decline of the nutritional quality of wucai during storage and could be used as an alternative method to prolong the storage time of postharvest wucai.

## CONFLICT OF INTEREST

The authors declare no conflict of interest.

## Supporting information

Fig S1

Fig S2

Fig S3

Fig S4

Fig S5

Fig S6

Fig S7

Fig S8

Fig S9

Fig S10

Fig S11

Fig S12

Fig S13

Table S1‐S4

## Data Availability

The data that support the findings of this study are available from the corresponding author upon reasonable request.
