# Supplementary figures and images for: The effect of exogenous 24‐epibrassinolide pretreatment on the quality, antioxidant capacity, and postharvest life of wucai (Brassica campestris L.)
Source: Food Sci Nutr. 2021 Jan 23;9(3):1323–35. doi: 10.1002/fsn3.2075 (PMC7958569; doi:10.1002/fsn3.2075)

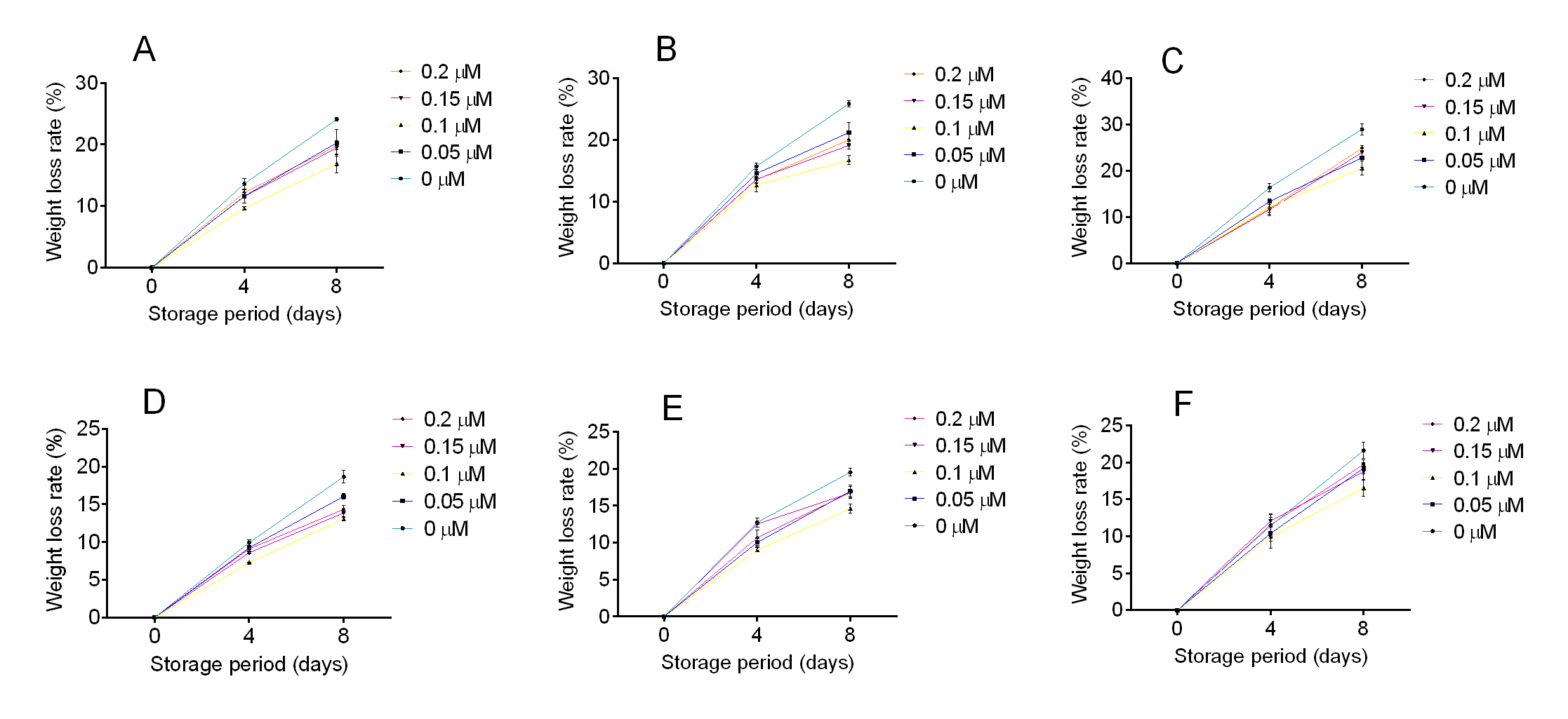

Supplement: Supplementary file 1 — Fig S1 [file FSN3-9-1323-s003.jpg]

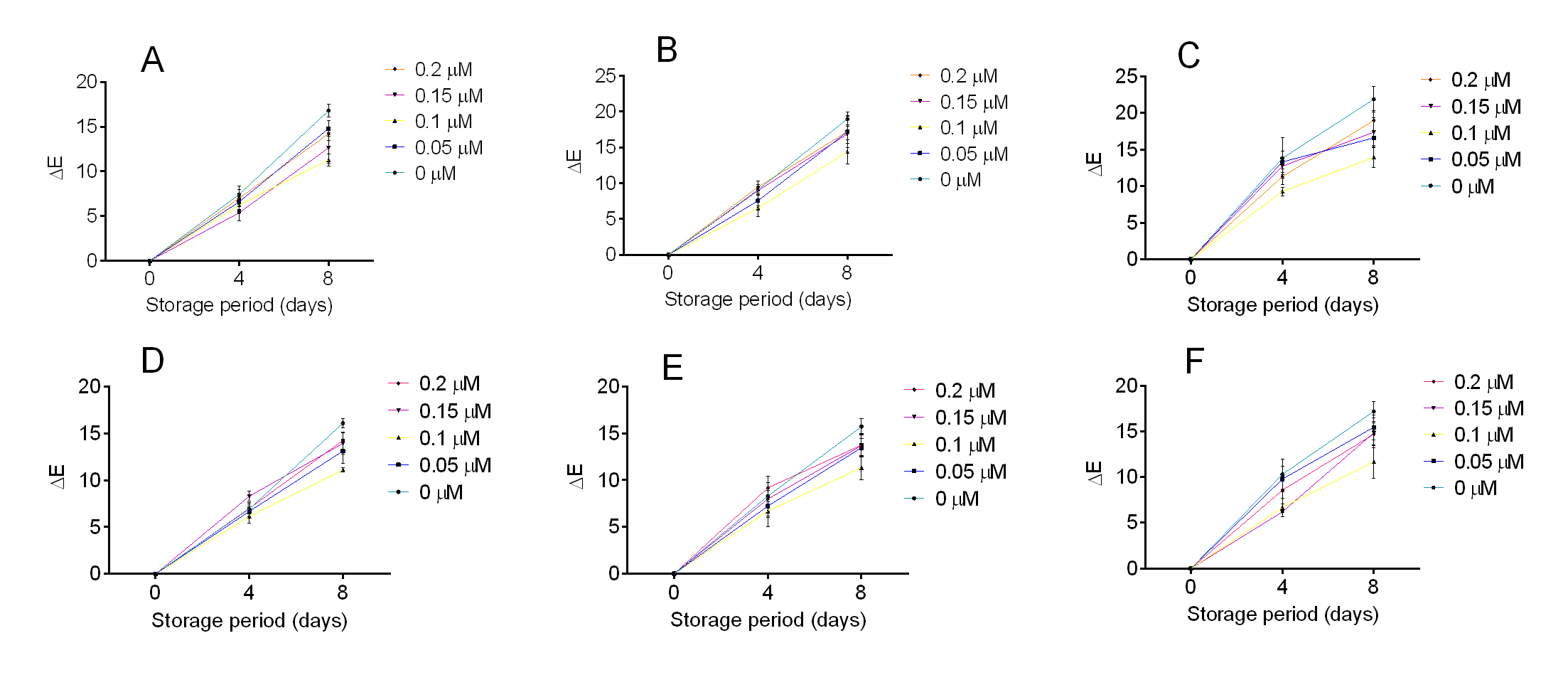

Supplement: Supplementary file 2 — Fig S2 [file FSN3-9-1323-s001.jpg]

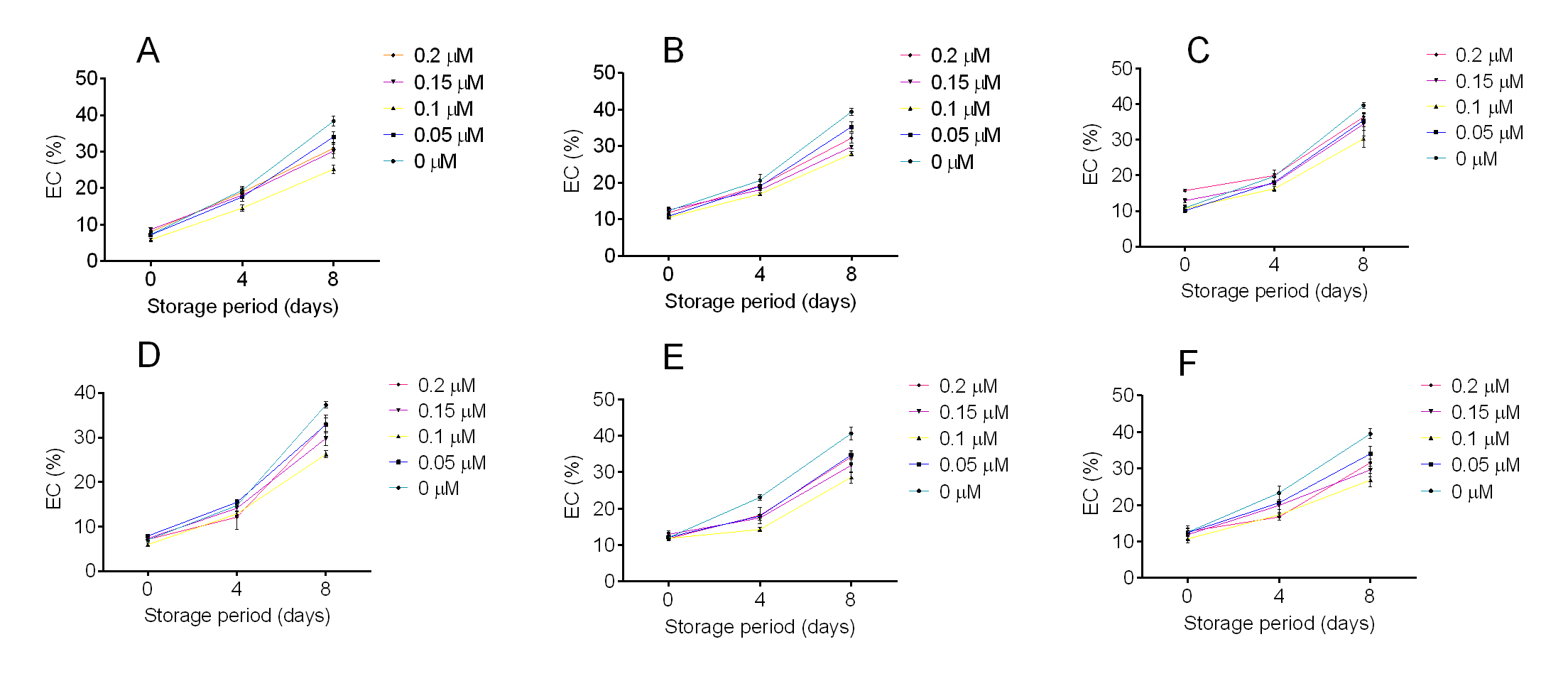

Supplement: Supplementary file 3 — Fig S3 [file FSN3-9-1323-s002.jpg]

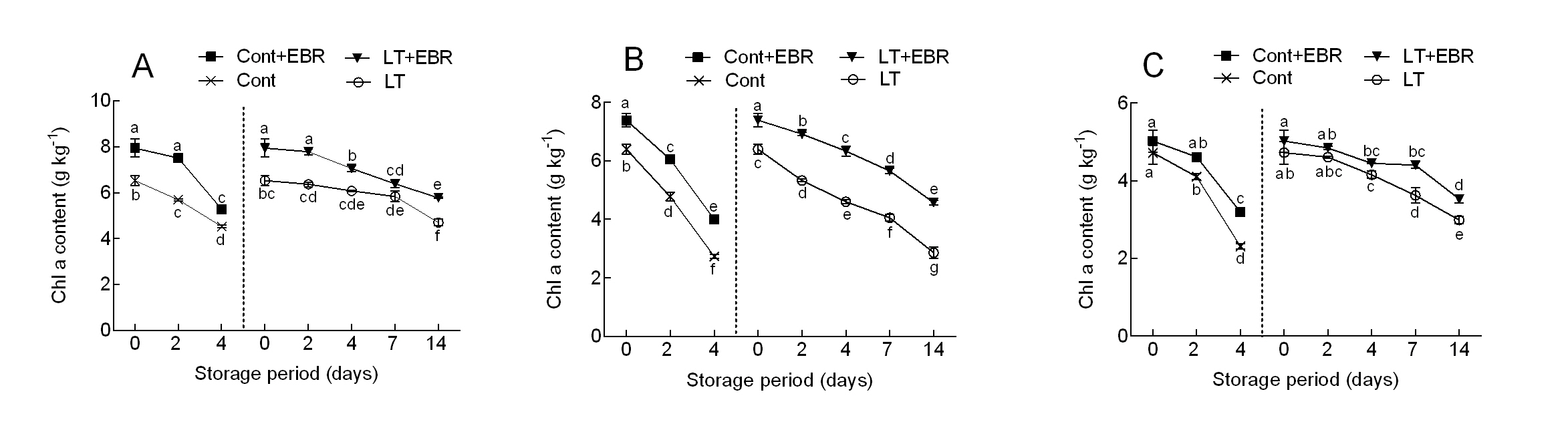

Supplement: Supplementary file 4 — Fig S4 [file FSN3-9-1323-s011.jpg]

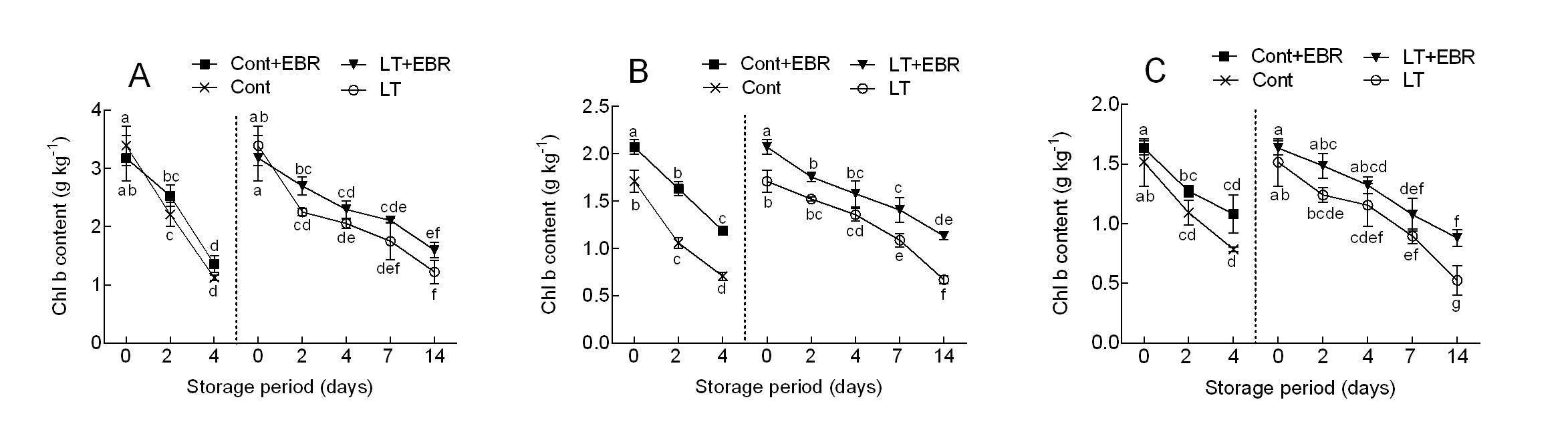

Supplement: Supplementary file 5 — Fig S5 [file FSN3-9-1323-s012.jpg]

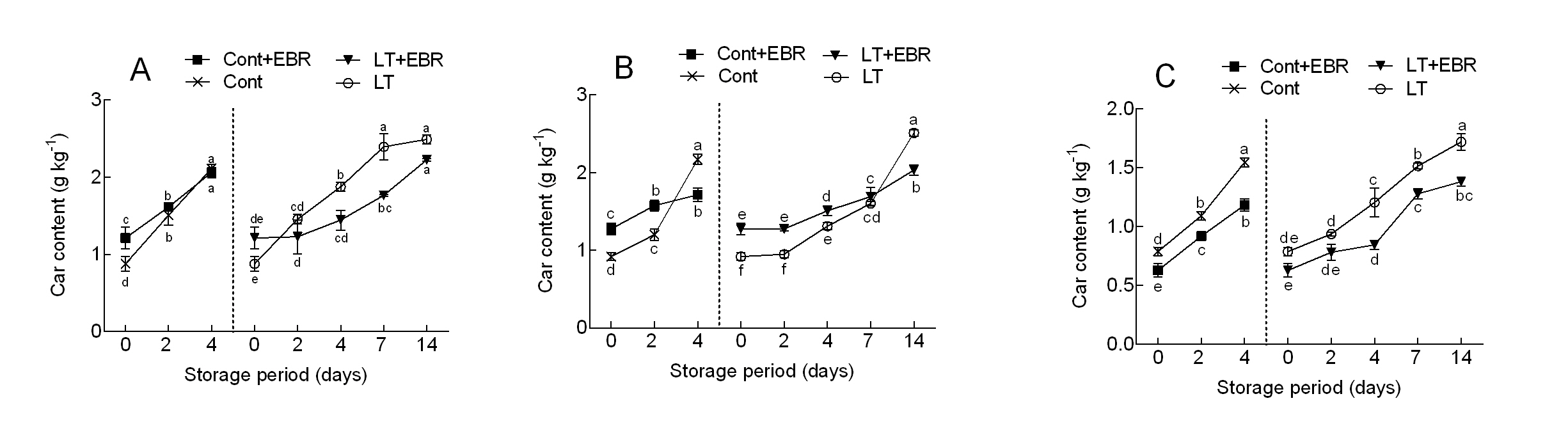

Supplement: Supplementary file 6 — Fig S6 [file FSN3-9-1323-s009.jpg]

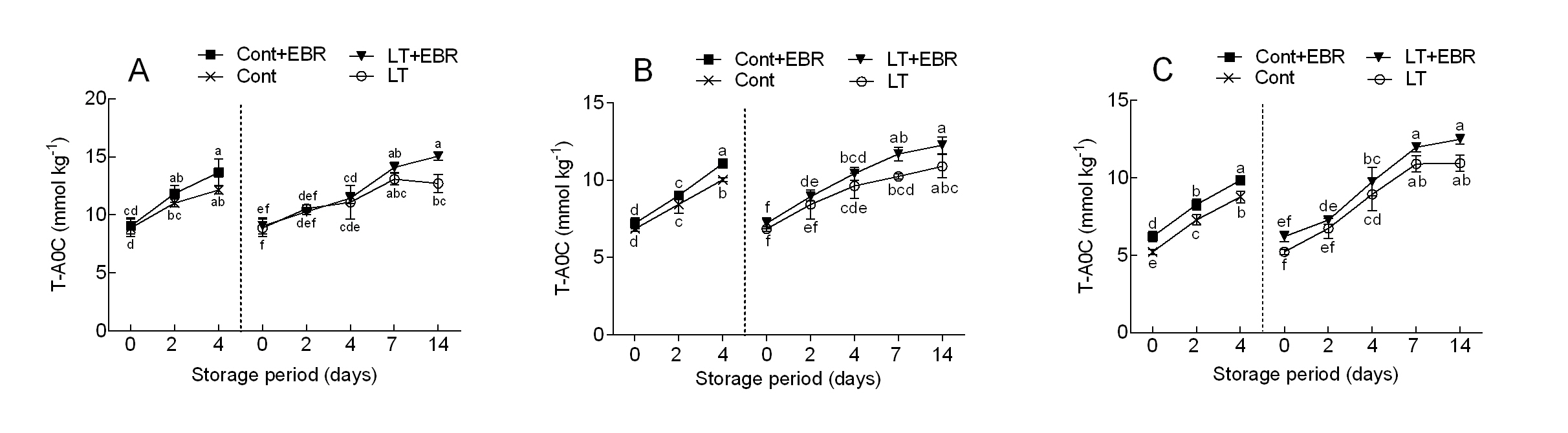

Supplement: Supplementary file 7 — Fig S7 [file FSN3-9-1323-s006.jpg]

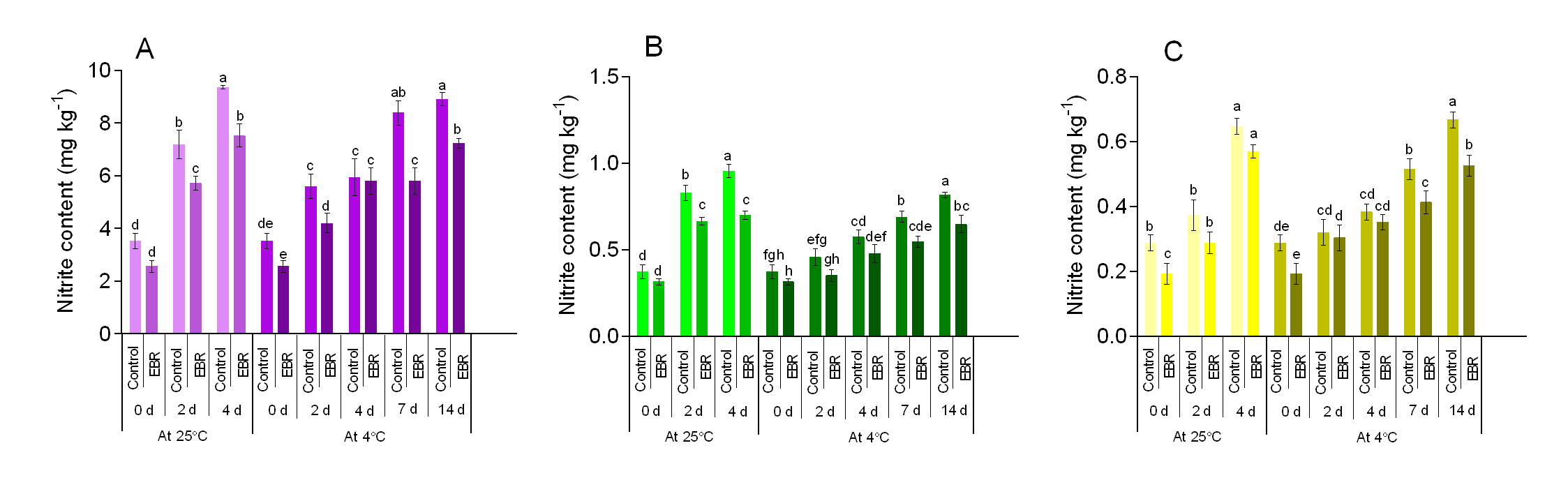

Supplement: Supplementary file 8 — Fig S8 [file FSN3-9-1323-s005.jpg]

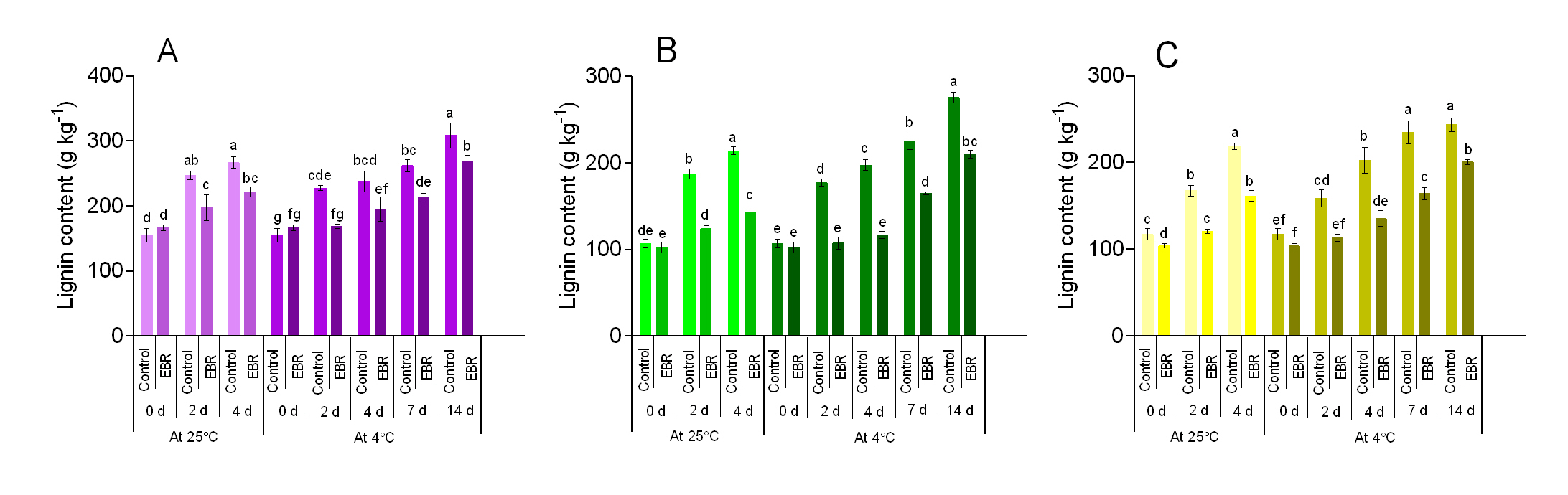

Supplement: Supplementary file 9 — Fig S9 [file FSN3-9-1323-s008.jpg]

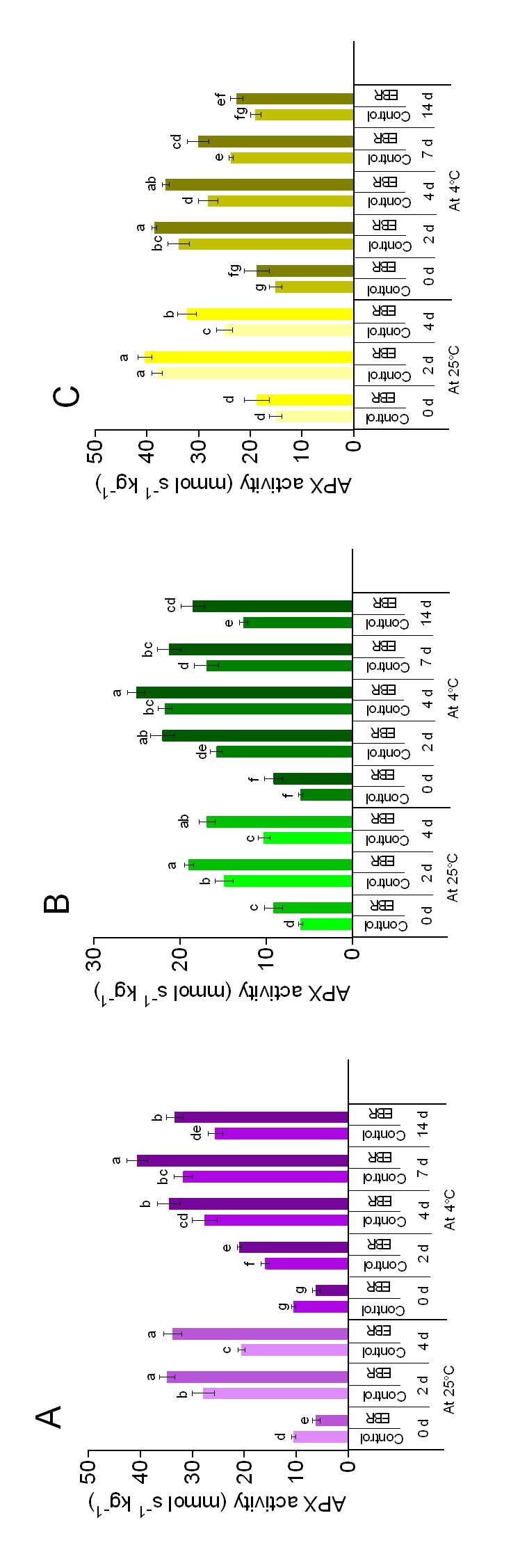

Supplement: Supplementary file 10 — Fig S10 [file FSN3-9-1323-s014.jpg]

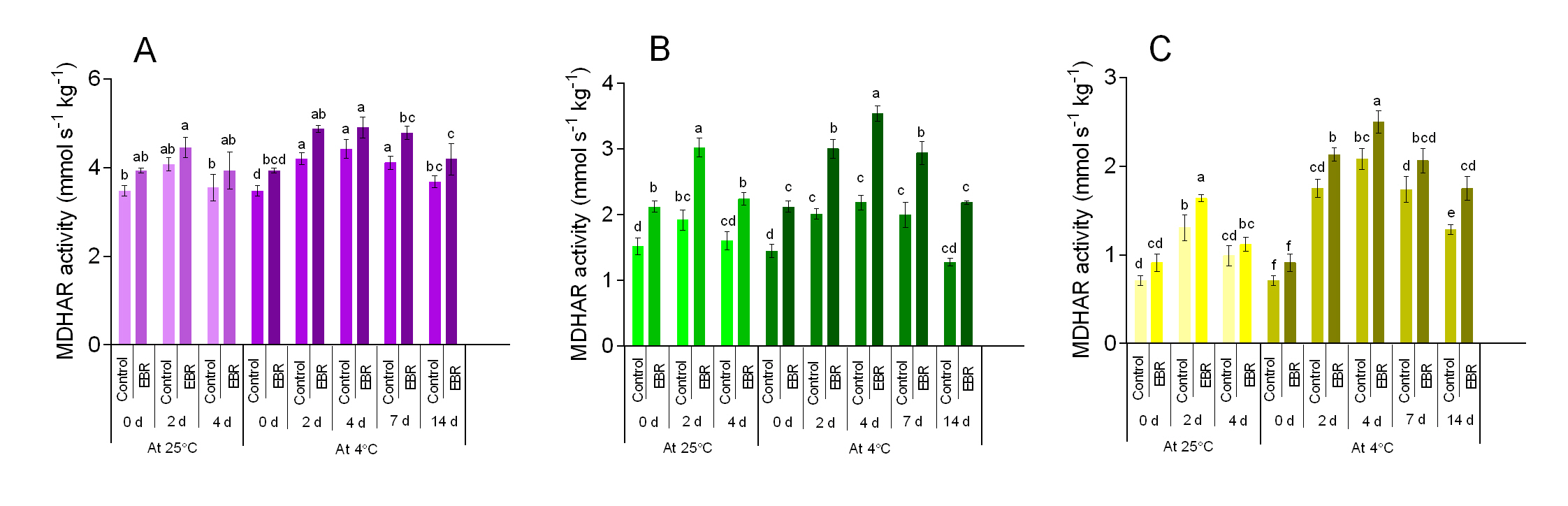

Supplement: Supplementary file 11 — Fig S11 [file FSN3-9-1323-s004.jpg]

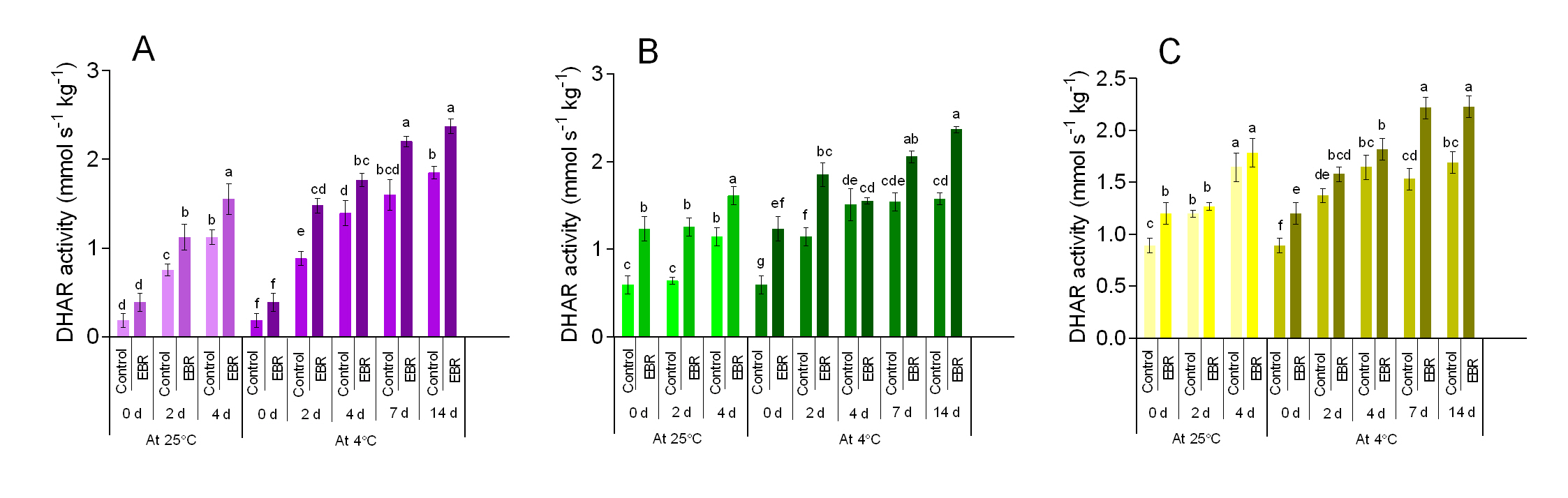

Supplement: Supplementary file 12 — Fig S12 [file FSN3-9-1323-s007.jpg]

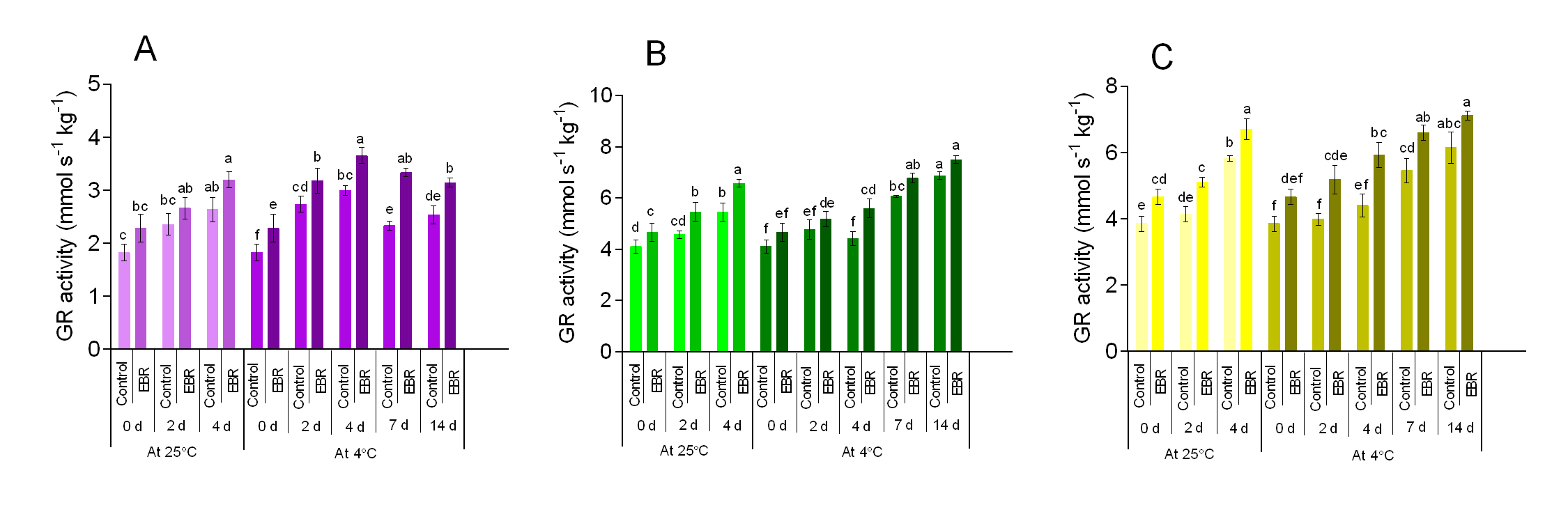

Supplement: Supplementary file 13 — Fig S13 [file FSN3-9-1323-s013.jpg]
