# Supplementary material for: The effect of exogenous 24‐epibrassinolide pretreatment on the quality, antioxidant capacity, and postharvest life of wucai (Brassica campestris L.)
Source: Food Sci Nutr. 2021 Jan 23;9(3):1323–35. doi: 10.1002/fsn3.2075 (PMC7958569; doi:10.1002/fsn3.2075)
Supplement: Supplementary file 14 — Table S1‐S4 [file FSN3-9-1323-s010.docx]

**Table S1.** Primer sequences

| **Gene** | **Forward primer** | **Reverse primer** |
| --- | --- | --- |
| *BnaActin* | 5' TGGGTTTGCTGGTGACGAT 3' | 5' TGCCTAGGACGACCAACAATACT3' |
| *APX1* | 5' GTGCCTGGACTTCAAACCCT 3' | 5' CTTCCTCGTCAGCAGCGT 3' |
| *APX3* | 5' CGTGAACTCCGTGCTCTCATCG 3' | 5' GGCACCATGACTGTACTCT 3' |
| *APX6* | 5' TCCAGAATCCCTCAATGCC 3' | 5' GAAGCCCAACCATACTCG 3' |
| *APXT* | 5' TAACATCTCTTACGCCGACT 3' | 5' CAGACAAGGCAACTATCTCC 3' |
| *AAO* | 5' CCTCAAACGCTAACGCAAC 3' | 5' CTTCACCATACCCAAGCACCC 3' |
| *MDHAR* | 5' ATGATCCAACATCTGCTAAACC 3' | 5' CCTCTACGCTCTCAACTACA 3' |
| *DHAR* | 5' AAGATCTCAGCAGCGGATTT 3' | 5' TTTGCGCCTCTGTATTCTTG 3' |

| **Amino acid (mg kg^-1^)** | **25°C** | **25°C+EBR** | **4°C** | **4°C+EBR** |
| --- | --- | --- | --- | --- |
| Nonessential amino acids |  |  |  |  |
| Aspartic | 266.2 | 996.48 | 1374.15 | 720.42 |
| Serine | 210.2 | 1166.86 | 1278.95 | 318.11 |
| Glutamic | 294.2 | 324.49 | 452.23 | 726.08 |
| Glycine | 150.2 | 257.09 | 271.57 | 140.8 |
| Alanine | 478.2 | 1746.43 | 1780.06 | 2238.18 |
| Tyrosine | 252.4 | 511.21 | 546.86 | 290.5 |
| Histidine | 310.4 | 424.96 | 495.15 | 265.41 |
| Arginine | 348.4 | 2561.04 | 2236.72 | 1129.28 |
| Proline | 430.2 | 2658.1 | 1440.38 | 5629.5 |
| Essential amino acids |  |  |  |  |
| Cysteine | 0 | 0 | 0 | 0 |
| Valine | 234.2 | 989.4 | 952.83 | 616.61 |
| Methionine | 138.4 | 34.99 | 21.3 | 17.34 |
| Threonine | 368.2 | 1925.35 | 2254.97 | 1080.18 |
| Isoleucine | 263.4 | 477.55 | 427.84 | 299.5 |
| Leucine | 262.4 | 560.73 | 488.26 | 371.09 |
| Phenylalanine | 330.4 | 910.58 | 1030.35 | 464.83 |
| lysine | 292.4 | 621.37 | 618.7 | 514.41 |
| Tryptophan | 0 | 0 | 0 | 0 |

**Table S2.** Analysis of free amino acids of PW-13 in four treatments at 4 d storage.

**Table S3.** Analysis of free amino acids of GW16-28 in four treatments at 4 d storage.

| **Amino acid (mg kg^-1^)** | **25°C** | **25°C+EBR** | **4°C** | **4°C+EBR** |
| --- | --- | --- | --- | --- |
| Nonessential amino acids |  |  |  |  |
| Aspartic | 886.9 | 1106.3 | 1140.76 | 797.28 |
| Serine | 327.01 | 1828.08 | 1594.32 | 704.26 |
| Glutamic | 980.35 | 1082.14 | 1312.15 | 1109.54 |
| Glycine | 160.82 | 394.85 | 342.66 | 202.44 |
| Alanine | 2376.22 | 1986.61 | 2496.8 | 2572.26 |
| Tyrosine | 267.94 | 820.36 | 793.3 | 579.83 |
| Histidine | 320.56 | 696.85 | 698.15 | 422.17 |
| Arginine | 1119.21 | 3209.67 | 3116.76 | 2460.48 |
| Proline | 4790.57 | 734.22 | 2615.83 | 3147.86 |
| Essential amino acids |  |  |  |  |
| Cysteine | 0 | 0 | 0 | 0 |
| Valine | 719.06 | 1422.15 | 1454.9 | 1008.8 |
| Methionine | 10.73 | 104.98 | 82.51 | 82.25 |
| Threonine | 1292.75 | 2199.34 | 2324.96 | 1409.9 |
| Isoleucine | 374.86 | 580.26 | 668.46 | 486.66 |
| Leucine | 426.09 | 753.8 | 737.19 | 648.91 |
| Phenylalanine | 547.46 | 1772.79 | 1312.58 | 1056.75 |
| lysine | 545.06 | 1156.14 | 869.84 | 1123.43 |
| Tryptophan | 0 | 0 | 0 | 0 |

**Table S4.** Analysis of free amino acids of YW15-8 in four treatments at 4 d storage.

| **Amino acid (mg kg^-1^)** | **25°C** | **25°C+EBR** | **4°C** | **4°C+EBR** |
| --- | --- | --- | --- | --- |
| Nonessential amino acids |  |  |  |  |
| Aspartic | 416.21 | 612.04 | 1173.71 | 651.85 |
| Serine | 505.17 | 790.55 | 1641.58 | 448.9 |
| Glutamic | 182.35 | 1078.67 | 1564.72 | 628.79 |
| Glycine | 136.34 | 231.31 | 332.72 | 240.46 |
| Alanine | 1615.28 | 2250.92 | 3198.59 | 2559.59 |
| Tyrosine | 416.24 | 668.8 | 727.84 | 351.93 |
| Histidine | 145.36 | 439.13 | 671.5 | 294.79 |
| Arginine | 1026.66 | 1970.22 | 3907.94 | 1716.64 |
| Proline | 3244.32 | 2206.27 | 2206.27 | 2535.25 |
| Essential amino acids |  |  |  |  |
| Cysteine | 0 | 0 | 0 | 0 |
| Valine | 649.13 | 1011.64 | 1473.78 | 863.64 |
| Methionine | 49.97 | 92.95 | 59.96 | 29.39 |
| Threonine | 942.92 | 1532.77 | 2508.04 | 1172.8 |
| Isoleucine | 296.25 | 541.07 | 824.51 | 435.34 |
| Leucine | 486.58 | 736.67 | 796.18 | 616.75 |
| Phenylalanine | 663.31 | 1016.1 | 1234.8 | 713.22 |
| lysine | 556.77 | 790.53 | 1005.4 | 658.32 |
| Tryptophan | 0 | 0 | 0 | 0 |
